# Resonator Based Switching Technique between Ultra Wide Band (UWB) and Single/Dual Continuously Tunable-Notch Behaviors in UWB Radar for Wireless Vital Signs Monitoring

**DOI:** 10.3390/s18103330

**Published:** 2018-10-04

**Authors:** MuhibUr Rahman, Mahdi NaghshvarianJahromi, Seyed Sajad Mirjavadi, Abdel Magid Hamouda

**Affiliations:** 1 Department of Electrical Engineering, Polytechnique Montreal, Montreal, QC H3T 1J4, Canada; muhibur.rahman@polymtl.ca; 2 Department of Electrical and Computer Engineering, McMaster University, Hamilton, ON L8S 4L8, Canada; 3 Health Technology Incubator, Jahrom University of Medical Sciences, 74148-46199 Jahrom, Iran; 4 Department of Mechanical and Industrial Engineering, College of Engineering, Qatar University, P.O. Box 2713 Doha, Qatar; seyedsajadmirjavadi@gmail.com (S.S.M.); hamouda@qu.edu.qa (A.M.H.)

**Keywords:** UWB behavior, switching of notched bands, miniaturized resonators, continuously tunable-band-notch behavior, tumor sensing

## Abstract

This paper presents a novel resonator that can switch and create three important behaviors within the same antenna using miniaturized capacitors. The resonator was integrated into conventional Ultra-Wide Band (UWB) antenna to achieve UWB and Single/Dual continuously tunable-notch behaviors. The Single/Dual notched was continuously tuned to our desired frequency band by changing the value of the capacitors. The antenna designed and fabricated to validate these behaviors had a compact size of 24 × 30.5 mm^2^, including the ground plane. The radiation patterns were very clean due to the placement of the proposed resonator in the special ground plane. Moreover, the presented novel resonator and switching technique was compared with the recently proposed resonators and their switching techniques. The prototype for the antenna was also developed in order to validate its performance in wireless vital signs monitoring. The presented miniaturized resonator based antenna was utilized for tumor sensing and simulations were provided in this regard. Moreover, the deployment of the proposed resonator based UWB antenna sensor in Pipeline Integrity Monitoring system was also investigated and discussed.

## 1. Introduction

Ultra-Wide Band (UWB) wireless communication has been on the spotlight since the last decade due to its speed and wide bandwidth. The frequency allocation for the range of 3.1 to 10.6 GHz is fixed for UWB wireless communication by the Federal Communication Commission (FCC). Currently, UWB transceivers need a miniaturized, planar, and multi-functional antenna that can support multi-operations utilizing a single structure [1].

For this purpose, different antennas have been designed and developed over time. Some of them target the miniaturization of antenna [2], while others focus on interference mitigation [3]. In [4], they achieved a triple band-notched behavior along with antenna miniaturization. Also, in [5] they removed all interfering frequency bands within the UWB spectrum and achieved Penta-notched behavior. Researchers also tried to achieve continuously tunable behavior [6], switchable behavior between narrow-band and wide-band [7], and reconfigurable notch band behavior [8,9]. All these techniques are limited up to two different behaviors from a single resonator.

In this paper, we have proposed a novel miniaturized resonator that has the ability to switch and create three important behaviors within the same structure using miniaturized capacitors. For validation purpose, this resonator was integrated into conventional UWB antenna [10], to achieve UWB and Single/Dual continuously tunable band-notch behaviors. Moreover, we have continuously tuned the Single/Dual notch in order to achieve the desired frequency band by changing the value of the capacitors. Antenna response was studied in these three different scenarios and important parameters, such as radiation pattern, antenna radiation efficiency, and antenna gain are provided for validation.

Currently, microwave sensors are used in tumor detection, brain stroke sensing, and heart failure sensing. These objectives can also be efficiently achieved by using antenna sensors as microwave imaging sensors, in order to detect the defective cells in the body. UWB antennas are advantageous in this regard since UWB signal offers better resolution as well as penetration properties.

Research has been carried out in this regard and different UWB band-notched antennas are implemented as an antenna sensor for different applications. In Reference [11], the authors developed a slotted tapered UWB antenna as a UWB antenna sensor for breast imaging. For tissue sensing applications, numerous types of UWB antennas as antenna sensors have been developed in past. Some common examples among them are microstrip patch antenna [12], planar monopole [13], and planar “dark eyes” [14].

Typically, the tissue or tumor is dipped in a liquid medium with comparable dielectric properties to that of tissue or tumor used for microwave scanning in the UWB frequency band [15]. The purpose of the liquid medium is that it minimizes reflections generated at the interface of skin, while increasing the signal penetration within the tissue or tumor. The optimization of the liquid medium has been given in detail in Reference [15]. In addition, comparisons of the different liquid medium have been addressed in Reference [16] with much more detail and with the pros and cons. However, selecting the proper liquid medium for the practical clinical experiment is a challenging task yet. Here, in our work, we have placed a planar microstrip continuously tunable dual notched UWB antenna in a shielded dielectric medium. The arrangement that differentiates the proposed antenna setup from previously reported techniques is:

(1) We completely eliminated the liquid coupling medium in our setup,
(2) All radiated power is directed towards the tissue or tumor,
(3) We blocked the interference from the surrounding as well as from the in-band signals that fall within UWB range.

These properties made the antenna beneficial to be used in the clinical environment as it maintains the sensor after the patient examination in the same state and thus reduces cost. Additionally, the second and most advantageous property is the detection and characterization of defective tumor cells in the body. The antenna designed for this purpose operate within the UWB frequency band ranging from 3.1 to 10.6 GHz, with a wide and tunable-notched band at 5.5 GHz, and also at 3.3 GHz. Moreover, the size of the antenna is very small compared to other antenna sensors in the literature. In addition, the resonators are very small and implemented in a partial ground plane which did not affect the radiation performance of the UWB antenna as an antenna sensor. The proposed UWB antenna sensor can be deployed as an integration with image processing technique, and in X-ray methods to detect cracks on the surface of the gas and oil pipelines. This is one of the challenging problems in the gas industry. Since the image processing technique and X-ray methods cannot detect the defects on above-ground pipelines, the application of the sensor can revolutionize the deployment of the devices used in Autonomous Robotic System for Pipeline Integrity Inspection.

The arrangement of the manuscript is carried out in the following manner: Section 2 deals with the design guidelines, proposed resonator analysis, simulation results from the proposed resonator and its integration in the conventional UWB antenna to achieve different behaviors. The measurement and validation of the proposed resonator integrated within the UWB antenna are explained in Section 3. The antenna sensor developed is dealt with and explained in Section 4. The proposed work is compared with the state-of-the-art designs and techniques in Section 5, which is followed by a conclusion.

## 2. Analysis, Design Guidelines, and Results

**Figure 1:**
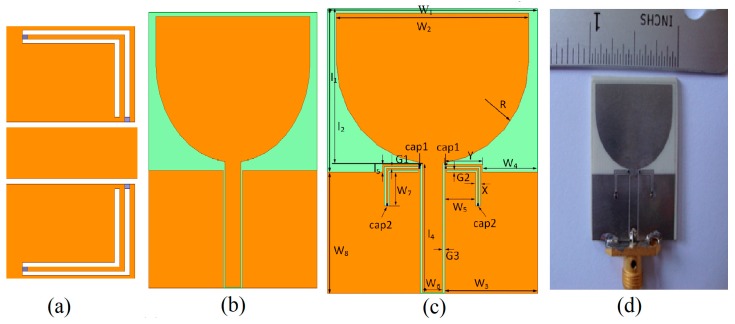
(**a**) Proposed miniaturized resonator; (**b**) reference UWB antenna; (**c**) UWB antenna with proposed resonator integrated; (**d**) prototype of the UWB antenna with proposed resonator integrated.

Figure 1a shows the miniaturized resonator implemented to achieve three important behaviors from a single structure. This resonator is advantageous in terms of (1) smallest size, (2) no effect on radiation performance of the resonator integrated antenna (almost comparable to that of reference antenna), and (3) achieving different important behaviors by changing the value of the capacitors. Figure 1b shows the reference UWB antenna that is simulated from 3.1 to 10.6 GHz, for comparison of radiation performance with the resonator integrated UWB antenna. Figure 1c shows the parameterized structure of the resonator integrated UWB antenna, while Figure 1d is the prototype for validation purpose only.

The substrate used for this purpose is Rogers RO4003 with a thickness of 1.5 mm and the relative dielectric constant of *ε_r_* = 3.38 having proposed dimensions of 24 × 30.5 mm^2^. Other parameters of the resonator integrated UWB antenna are mentioned in Table 1. In the manuscript, we have utilized four capacitors according to the American Technical Ceramics (ATC) design kit, as labeled as Cap1 and Cap2 in Figure 1c. Cap1 and Cap2 are varied which changes the behavior of the antenna and make the resonator advantageous to achieve three different behaviors. Different combination and variations for the capacitors are studied and their behaviors are presented in the manuscript.

### 2.1. Analysis of the Proposed Resonator

The proposed resonator can be analyzed for its corresponding start and stop frequencies which will be utilized for simulation and measurements. The effective permeability, *µ_eff_*, and effective permittivity, *ε_eff_*, can be calculated by utilizing the following equations in sequence [7]:

(1) $$\Gamma =k\pm \sqrt{k^{2}-1}$$

(2) $$k=\frac{S_{11}{}^{2}-S_{21}{}^{2}+1}{2S_{11}}$$

(3) $$Z_{eff}=\sqrt{\frac{\mu_{eff}}{\varepsilon_{eff}}}=\left(\frac{1+\Gamma }{1-\Gamma }\right)\frac{Z^{TL}}{Z_{a}{}^{TL}}$$

(4) $$n=n^{\prime }-jn^{\prime \prime }=\sqrt{\varepsilon_{eff}\mu_{eff}}=\pm \frac{c}{j\omega l}\cos h^{-1}\left(\frac{1-S_{11}{}^{2}-S_{21}{}^{2}}{2S_{21}}\right)$$

(5) $$\varepsilon_{eff}=\varepsilon_{eff}{}^{\prime }-j\varepsilon_{eff}{}^{\prime \prime }=\frac{n}{Z_{eff}}$$

(6) $$\mu_{eff}=\mu_{eff}{}^{\prime }-j\mu_{eff}{}^{\prime \prime }=n\times Z_{eff}$$

where Γ represents the reflection coefficient, $Z^{TL}$ is the characteristic impedance of the reference transmission line while $Z_{a}^{TL}$ is the characteristic impedance of the transmission line in case of air-filled, *l* represents the effective length of the resonator, and *n* signifies the refractive index.

The overall length of the proposed resonator is almost λg/2 (where, λg is the guided wavelength) while Coupling coefficient is calculated as $n=\sqrt{\frac{Z_{\left(cpw\right)}}{Z_{os}}}$, where *Z_(cpw)_* is the characteristics impedance of Co-Planar Waveguide (CPW) transmission line and *Z_os_* is the characteristics impedance of the slot line resonator. The reflection (S_11_) and transmission coefficient (S_21_) is determined by considering the proposed resonator as a two-port matched filter. When the capacitor value (Cap1 = 0.1 pF), the resonator behaves as an open circuit and creates a fundamental resonance at 3.8 GHz and second resonance at 7.6 GHz. By increasing Cap1, both the fundamental and second resonance can be shifted to lower frequencies accordingly.

### 2.2. Continuosly Dual Band-Notched Behavior of the Resonator

**Figure 2:**
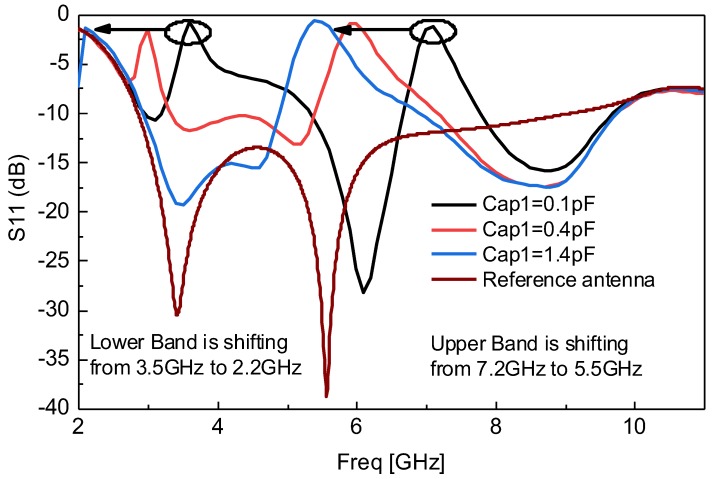
Continuously tunable band-notch behavior of resonator by varying Cap1 only.

Figure 2 shows the simulated response of continuously dual band-notched behavior, achieved from the proposed resonator integrated in conventional UWB antenna correlated with the reference UWB antenna response. It can be seen from Figure 2 that the resonator has the ability to continuously tune the notched bands by varying Cap1 only. The upper notched band is set at 7.2 GHz while the lower notched band is set at 3.5 GHz, at Cap1 = 0.1 pF. It is observed and shown that by changing the Cap1 value from 0.1 pF to 1.4 pF the upper and lower notched bands are continuously changed to 5.5 GHz and 2.2 GHz respectively as shown in Figure 2. Cap2 is chosen as fixed having value of 0.1 pF. This is one of the important behaviors which can be achieved from the proposed resonator instead of designing multiple resonators for different frequencies and different filtering bands.

### 2.3. Switching between Dual Notched and Single Notched Behavior

**Figure 3:**
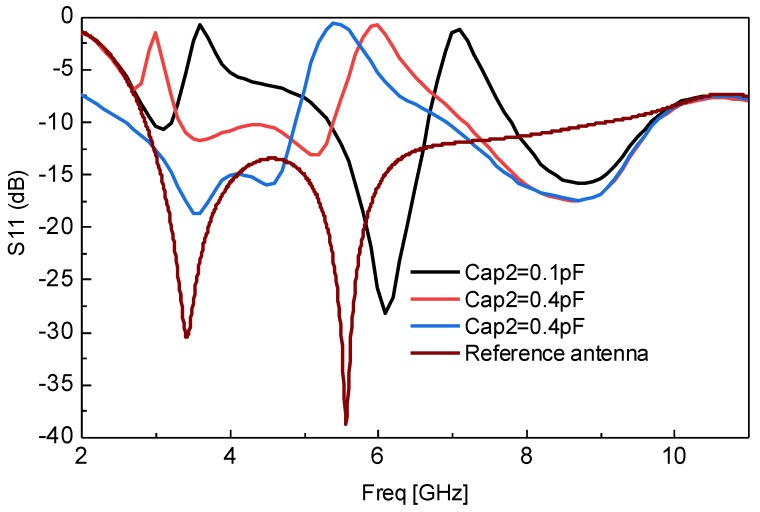
Switching between dual and single notched behavior by varying Cap2 only.

Figure 3 shows the switching of dual notched behavior to a single WLAN (Wireless Local Area Network) notched response correlated with the reference UWB response. It can also be seen from Figure 3 that the proposed resonator has the ability to continuously tune dual notched bands as well as transforming it into the single notched band by varying Cap2 only. The upper notched band is set at 7.2 GHz while the lower notched band is set at 3.5 GHz, at Cap1 = 0.1 pF. It is observed and shown that by changing the Cap2 value from 0.1 pF to 1.4 pF, the upper notched bands are continuously changed and tuned to 5.5 GHz while the lower notched band is shifted and diminished at Cap2 = 1.4 pF. This is another important behavior of the proposed resonator which can be used for single WLAN notched band tuning, as well as switching between dual notched and single notched response. However, it must be noted that the single notched behavior is only achieved for the lower values of the upper band because when the upper resonance is about 7.2 GHz, it is impossible to achieve a single notched behavior.

### 2.4. Switching between Dual Notched and Wideband Behavior

**Figure 4:**
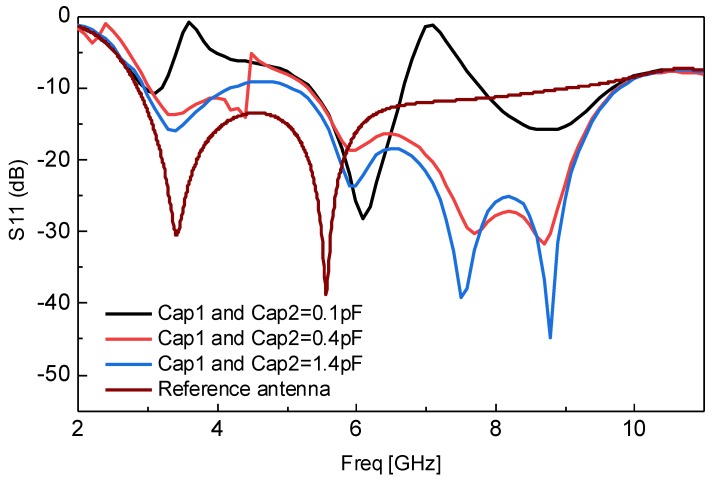
Switching between UWB and dual notched UWB by simultaneously changing Cap1 and Cap2 of the proposed resonator.

The third important behavior of the proposed resonator in its implemented application is highlighted in Figure 4. It can be seen from Figure 4 that the response of the antenna can be switched continuously between dual notched UWB and simple UWB behavior by changing Cap1 and Cap2, simultaneously. This behavior makes the antenna advantageous by designing UWB, as well as band-notched UWB antenna in the same structure. Previously, researchers have designed antennas only for UWB operation or for band-notched UWB operation. Due to this behavior, we have achieved multi-functions from a single structure using the proposed miniaturized resonator.

## 3. Measurements

**Figure 5:**
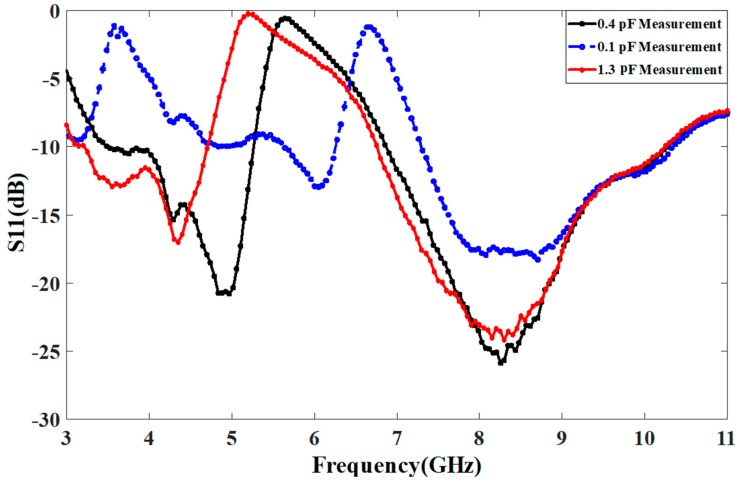
Measured response of the antenna with the proposed resonator integrated (changing Cap1).

**Figure 6:**
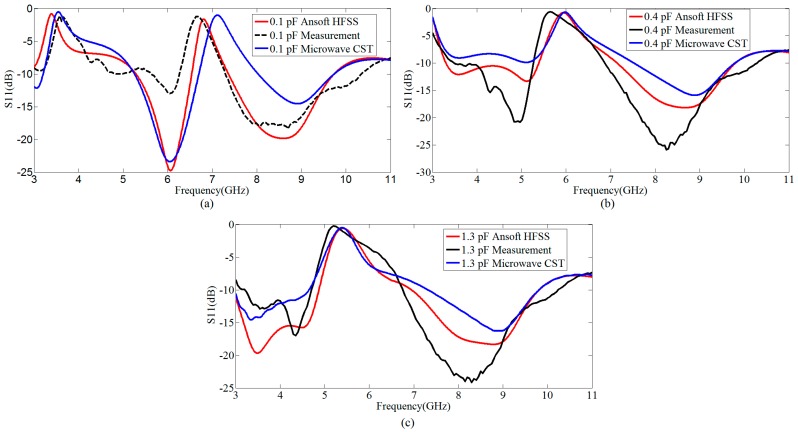
Simulated versus measured results of the proposed resonator based switchable UWB band-notched antenna; (**a**) at 0.1 pF; (**b**) at 0.4 pF; (**c**) at 1.3 pF.

The integration of the proposed resonator is carried out within the conventional UWB antenna and the prototype is developed as shown in Figure 1d. The antenna integrated with the proposed resonator is measured at different values of the capacitor. In measurement, the validation is only performed in case of varying Cap1 only and the measured correlated results at different capacitor values are shown in Figure 5. The value of Cap2 is fixed at 0.1 pF. The measurement of the antenna is performed using HP-8720 Vector network analyzer. Figure 6 shows the simulated versus measured response of the proposed resonator based switchable UWB band-notched antenna at Cap1 = 0.1 pF, 0.4 pF, and 1.3 pF, having Cap2 fixed at 0.1 pF. The measured results are correlated with simulations taken from two EM (Electromagnetic) simulators CST (Computer Simulation Technology) as well as HFSS (High Frequency Structure Simulator). The measured results support the simulations.

The difference between the measured results of HFSS and CST are due to different computational techniques of both EM solvers. HFSS is based on Finite Element Method (FEM), which is more accurate for designing antennas, while CST is based upon Finite Integration Technique (FIT) and is also popular among antenna designers due to ease in simulations. However, the results of both simulators are not exactly same because of different computational techniques involved. We have compared this behavior of two simulators for our design also, and presented the results in Figure 6.

It is very important to note that we have used capacitors that are according to ATC design kit, and varied the capacitors during measurement to make the antenna response switchable. Although we can use the varactor for this purpose as well, the ATC capacitors are very exact with tolerance, and we have checked it for three different values. This helps us to compare measurement results more exactly with simulation results. Even in reconfigurable antennas or switchable antennas with MEMS (Micro-Electro-Mechanical Systems), researchers are using this approach and technique. For further use in portable devices, we can use the same antenna with varactor high Q that will give almost the same response as with ATC capacitors.

### Radiation Performance of the Resonator Integrated Dual Notched Antenna

**Figure 7:**
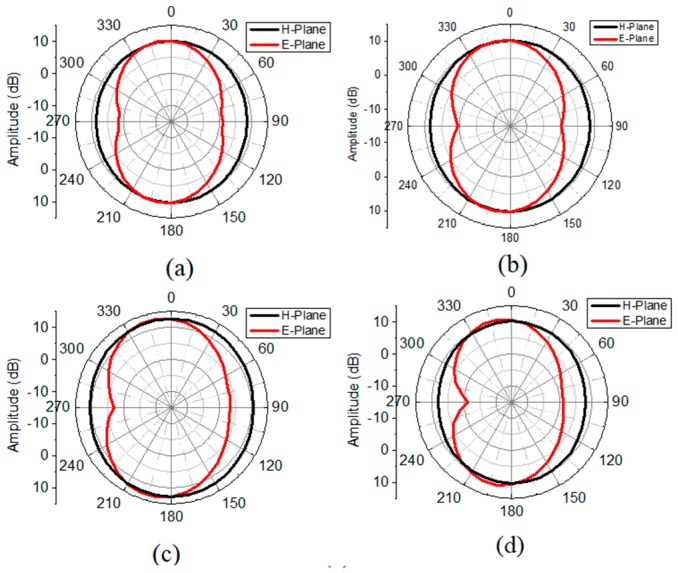
Radiation patterns of the proposed resonator based continuously tunable dual band-notched antenna at Cap1 = 0.1 pF (**a**) 3.5 GHz (simulated); (**b**) 5.9 GHz (measured); (**c**) 8.1 GHz (measured); (**d**) 10 GHz (simulated).

**Figure 8:**
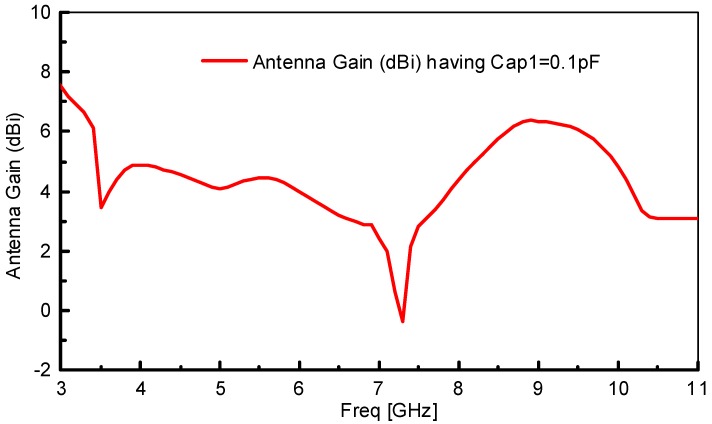
Simulated gain (dBi) of the proposed resonator based continuously tunable dual band-notched antenna at Cap1 = 0.1 pF.

The radiation performance of the resonator integrated dual notched antenna at frequencies of 3.5, 5.9, 8.1 and 10 GHz are displayed in Figure 7. The radiation pattern at 3.5 and 10 GHz is taken from simulations, while it is measured at 5.9 and 8.1 GHz. Radiation patterns are simulated and measured having Cap1 and Cap2 = 0.1 pF. Clean and consistent radiation patterns are obtained even at high frequencies due to the size and placement of the proposed resonator in the ground plane instead of placing in the radiator. This phenomenon further makes the resonator advantageous by creating notched bands without degrading radiation performance. There arise ripple at 10 GHz which is due to distortion in the phase distribution of the electric field and the increase in the magnitude of higher order harmonic modes at the higher frequency. In addition, the simulated gain (dBi) of the proposed resonator based continuously tunable dual band-notched antenna at Cap1 = 0.1 pF is provided in Figure 8.

## 4. Proposed Resonator Based Antenna Application in Breast Tumor Detection

**Figure 9:**
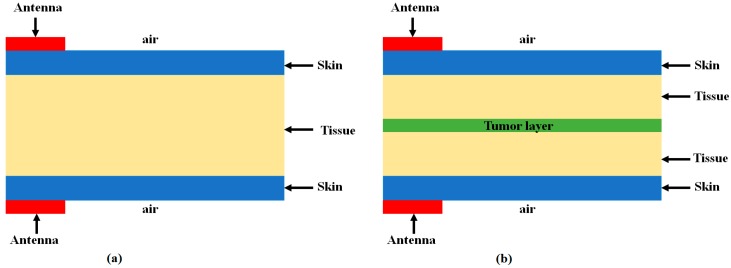
Model developed for investigating received signal responses with biological organic stacked layers (cross view); (**a**) Model developed without tumor layer; (**b**) Model developed with tumor layer [17].

The proposed resonator based developed antenna is successfully utilized as a UWB antenna sensor, that has the capability to detect defective cells in the body without any liquid medium. To develop the designed antenna as an antenna sensor, it is investigated in detail with the biological organic layers using the 3D EM simulator, CST Microwave studio suite. The setup is made in such a way that the developed antenna sensor is placed in contact with the biological layers with and without defective cells [17]. The presented developed models are shown in Figure 9a,b for the above cases. This is an inhomogeneous developed dielectric medium that has the capability to act as a skin, tissue, and tumor. The conductivity and permittivity of tissue, skin, and tumor can be given from Table 2, which is analyzed and calculated in [13].

The proposed antenna sensor is excited with the first-fifth order Gaussian pulse generated in CST. By transmitting the short UWB pulses with the proposed antenna sensor, we can detect the received strength of the transmitted pulse by placing another proposed antenna sensor in contact with, or at a certain distance from, the biological stacked organic layers. Firstly, the transmitted pulse is passed from the non-defective layer to visualize its received strength. Secondly, the transmitted pulse is then passed from the body having suspect of defective cells and its received strength is observed. By comparing both cases, we can judge whether there are tumors in the body or not. This is performed by observing the received waveform in two cases: (1) No tumor model as in Figure 9a and (2) tumor model as in Figure 9b.

It should be noted that this model only provides an insight into tumor layers, not exact geometry (having conductivity, mass density, and dielectric constant) comparable to tumors analyzed. Moreover, this technique helps us to show that the antenna possesses the capability of detecting tumors, but not by guarantee. In addition, it has been investigated that the most critical parameters are dielectric constant, conductivity, and mass density, as analyzed in Reference [13]. The thickness of skin, tissue, and tumor layer has a very slight effect on the amplitude response.

**Figure 10:**
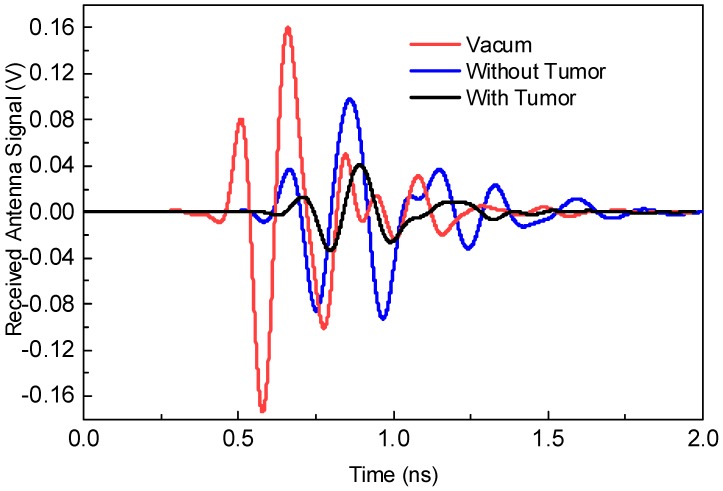
Received signal responses when both antenna sensors are positioned in contact with biological stacked organic layers.

Figure 10 shows the first experiment with an amplitude of the received waveform in three different cases having vacuum, no tumor layer, and with tumor layer. In this experiment, the antenna sensors are placed in contact with the biological stacked organic layers. By comparing the three responses, it can be easily judged that the presence of the tumor layer considerably decreases the strength of the received waveform as can be realized from Figure 10.

**Figure 11:**
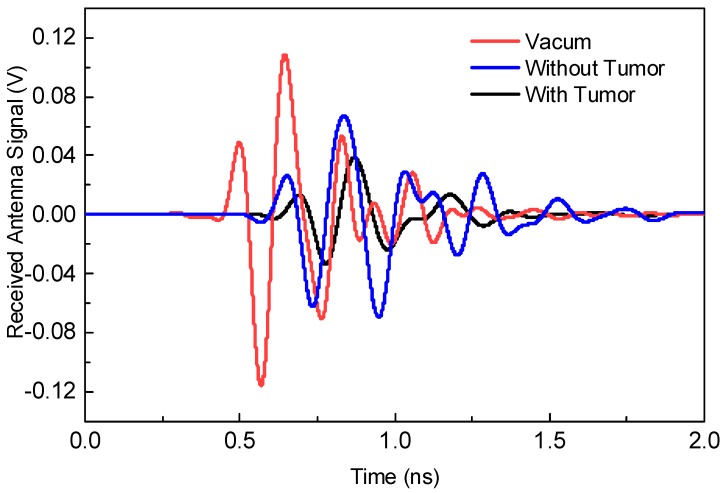
Received signal responses when both antenna sensors are positioned 15 mm away from the biological stacked organic layers.

Figure 11 shows the second experiment with an amplitude of the received waveform when antenna sensors are placed at a distance of 15 mm from the biological stacked organic layers. Also, by comparing the responses here, it can be judged that the presence of tumor layer considerably decreases the strength of the received waveform.

By comparing the response of both experiments, it can be visualized that in experiment 1, the received signal strength is stronger and greater as compared to experiment 2. This is because the distance between the two sensors in experiment 1 is smaller compared to experiment 2, where other loss effects may also take place. It is also clear that the placement of biological stacked organic layers i.e. tissues, skin, and tumor decrease the received waveform strength due to losses on the surface and multiple reflections from each layer during transmission. Due to different dielectric properties as shown in Table 2, the losses from different layers vary differently.

### Implementation of Proposed Antenna in Microwave Imaging Application

**Figure 12:**
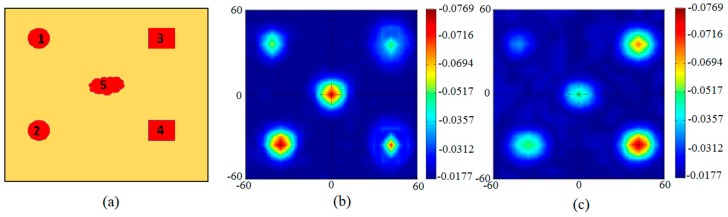
(**a**) Proposed phantom model with selected tumor geometries. (**b**) Calibrated S21 (magnitude) image from 2D scanning at 5 GHz (simulated). (**c**) Calibrated S21 (magnitude) image from 2D scanning at 8 GHz (simulated).

The proposed resonator based antenna performance in a microwave imaging sensing application is also checked and the results are provided. Aperture scanning method [18,19,20] is utilized to detect different tumors in a body using microwave imaging technique. The breast phantom model developed in the previous section has been utilized and five different tumor geometries have been placed for the purpose of testing. Tumor 1 and 2 possess spherical shape (radius having 3 mm), Tumor 3 and 4 possess a cuboid shape (face area = 6 mm), and Tumor 5 has an irregular geometry as shown in Figure 12a. The performance of the reported antenna for this scanning method is examined by means of the CST EM Solver. The S21 were obtained over the whole phantom model with a 5 mm spatial sampling rate. The images developed from calibrated S21 at 5 GHz and 8 GHz are shown in Figure 12b,c, respectively. Tumors were detected as shown in these Figures, however their exact shapes are not clear due to their miniaturized size. The tumors shapes can be made identifiable in future by applying strong image processing algorithm.

The imaging setup uses two antennas, one to send a UWB signal and the other to receive it, to accomplish a 2D scan in the (*y*, *z*) plane. The breast is compressed between the antennas, and the antennas move together in fixed steps until the breast is completely scanned. The duration of the signal is in the nanosecond regime at a given breast location and therefore the heating effect can be neglected. The image was formed as follows: First, the phantom without tumors was placed between the antennas and the transmission S-parameter between the two antennas at each position (*y*, *z*) was recorded as $S_{21}{}^{back}\left(y,z\right)$. Then the same phantom with many tumors was placed between the two antennas to get the simulated transmission S-parameter $S_{21}{}^{sim}\left(y,z\right)$. The image was acquired by subtracting transmission S-parameter of phantom with no tumors from transmission S-parameter of phantom with tumors at each position then taking the absolute value from the Equation (7):

(7) $$\left|S_{21}{}^{image}\left(y,z\right)\right|=\left|S_{21}{}^{sim}\left(y,z\right)-S_{21}{}^{back}\left(y,z\right)\right|$$

**Figure 13:**
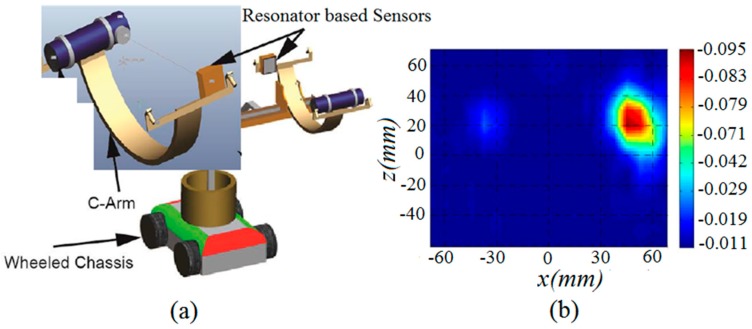
(**a**) Deployment of the proposed UWB antenna resonator based sensor in Pipeline Integrity Monitoring system; (**b**) calibrated S21 (magnitude) image from 2D scanning at 10 GHz for the industrial steel pipeline with two breaches on both sides (simulation).

The second application of the proposed antenna is also developed and implemented in pipeline monitoring system. The implementation of the proposed resonator based UWB antennas sensor in Proactive Pipeline Integrity Monitoring system is shown in Figure 13, which is a canonical model for breach detection in pipeline. Simulation has been performed based on topology in Figure 13, where an aperture scanning method is used for industrial steel pipeline breach detection. For simulation, an industrial steel pipeline is selected based on its properties [21,22], with a breach on both sides, and placed at distance of 5 mm from both antenna sensors. It is seen from the simulations that the exact position is judged in XZ-coordinates, however the breach size is not clear. It can be further investigated in future and made identifiable by applying some image processing technique. This application of the antenna sensor can revolutionize the deployment of the devices used in Autonomous Robotic System for Pipeline Integrity Inspection, in combination with the image processing technique and X-ray method in future.

## 5. Comparison with Recently Proposed Resonators Used for Notching and Switching Purposes

The proposed resonator functionalities are compared with the recently reported resonators in the literature. The developed application from the proposed resonator is also compared with their applications such as antennas or filters. The comparison shows that the proposed resonator is advantageous in terms of maximum behaviors within the same structure, small size, and having clean radiation performance in its application. Table 3 provides a detailed comparison, which is helpful in understanding the novelty of the proposed resonator.

## 6. Conclusions

This paper presents a novel resonator that can switch and create three important behaviors within the same antenna structure, with the addition of miniaturized four capacitors. The resonator has been successfully integrated into conventional UWB antenna and achieved UWB and Single/Dual continuously tunable-notch behaviors. The Single/Dual notch behavior achieved can also be continuously tuned to our desired frequency band by changing the value of the capacitors. For this purpose, the antenna is designed and fabricated to validate these behaviors having a compact size of 24 × 30.5 mm^2^ including the ground plane. The radiation patterns are very clean due to the placement of the proposed resonator in the special ground plane. Moreover, the presented resonator and its functionalities are compared with the recently proposed resonators and its superiority over other techniques is analyzed. The prototype for the antenna is also developed in order to validate its performance as a useful sensor for wireless signs monitoring applications. The presented miniaturized resonator based antenna is successfully simulated as an antenna sensor for tumor detection. Moreover, the deployment of the proposed UWB antenna resonator based sensor in Pipeline Integrity Monitoring system is also investigated.

## Figures and Tables

**Table 1:** Dimensions (millimeters) of the resonator integrated Ultra Wide Band (UWB) antenna.

Parameters	l_1_	l_2_	l_4_	T	H	X
Value (mm)	17.5	16	13.865	0.017	1.5	0.6
Parameters	Y	W_1_	W_2_	W_3_	W_4_	W_5_
Value (mm)	4.3	24	22	10.6	6.3	3.5
Parameters	W_6_	W_7_	W_8_	G1	G2	R
Value (mm)	2.3	3.6	13	0.25	0.5	11

**Table 2:** Dielectric properties of the stacked organic layers, i.e., tissue, skin, and tumor [13].

Type	Conductivity (S/m)	Mass Density (Kg/m^3^)	Dielectric Constant	Height (mm)
Skin	3	1010	37	0.10
Tissue	7	1040	50	0.05
Tumor	5	1041	68	0.30

**Table 3:** Performance comparison of the proposed technique with other reported techniques.

Reference	Resonator Functionalities	Developed Antenna/Filter Size (mm^2^)	Remarks
[23]	UWB behavior using SCRLH (Simplified Composite Right/Left-Handed) resonator	34 × 22	(i) Single wideband behavior is achieved and implemented as a BPF (Bandpass Filter)
[24]	Can be used for UWB operation Can be used for single/dual notching	30.3 × 24.8	(i) Two different behaviors are achieved from a single resonator using PIN diodes (ii) Clean Radiation performance
[25]	Dual notched UWB switching from two slotted resonators	40 × 40	(i) Single behavior is achieved from two resonators (ii) Deteriorated radiation performance
This Work	Can be used for UWB operation Can be used for single/dual notching Can be used for switching b/w above two behaviors	24 × 30.5	(i) Three behaviors from a single resonator (ii) Implemented as an antenna sensor for tumor detection (iii) Clean radiation performance
